# Adiponectin and leptin exert antagonizing effects on proliferation and motility of papillary thyroid cancer cell lines

**DOI:** 10.1007/s13105-021-00789-x

**Published:** 2021-02-15

**Authors:** Ersilia Nigro, Francesca Maria Orlandella, Rita Polito, Raffaela Mariarosaria Mariniello, Maria Ludovica Monaco, Marta Mallardo, Anna Elisa De Stefano, Paola Lucia Chiara Iervolino, Giuliana Salvatore, Aurora Daniele

**Affiliations:** 1 Dipartimento di Scienze e Tecnologie Ambientali Biologiche Farmaceutiche, Università degli Studi della Campania, “Luigi Vanvitelli,”, Via G. Vivaldi 42, 81100 Caserta, Italy; 2 CEINGE - Biotecnologie Avanzate S.c.a.r.l., Via Gaetano Salvatore 486, 80145 Napoli, Italy; 3 IRCCS SDN, Via Emanuele Gianturco 113, 80143 Napoli, Italy; 4 Dipartimento di Scienze Motorie e del Benessere, Università degli Studi di Napoli “Parthenope,”, Via Medina 40, 80133 Napoli, Italy; 5 Dipartimento di Scienze Biomediche Avanzate, Università degli Studi di Napoli “Federico II,”, Via Pansini 5, 80131 Napoli, Italy

**Keywords:** Adiponectin, Leptin, Thyroid cancer, Proliferation, Motility

## Abstract

Adiponectin (Acrp30) and leptin, adipokines produced and secreted mainly by the adipose tissue, are involved in human carcinogenesis. Thyroid carcinomas are frequent endocrine cancers, and several evidences suggest that they are correlated with obesity. In this study, we first analyzed the expression levels and prognostic values of Acrp30, leptin, and their receptors in thyroid cancer cells. Then, we investigated the role of Acrp30 and leptin in proliferation, migration, and invasion. We found that Acrp30 treatment alone inhibits cell proliferation and cell viability in a time and dose-dependent manner; leptin alone does not influence thyroid cancer cells (BCPAP and K1) proliferation, but the combined treatment reverts Acrp30-induced effects on cell proliferation. Additionally, through wound healing and Matrigel Matrix invasion assays, we unveiled that Acrp30 inhibits thyroid cancer cell motility, while leptin induces the opposite effect. Importantly, in the combined treatment, Acrp30 and leptin exert antagonizing effects on papillary thyroid cancer cells’ migration and invasion in both BCPAP and K1 cell lines. Highlights of these studies suggest that Acrp30 and leptin could represent therapeutic targets and biomarkers for the management of thyroid cancer.

**Supplementary Information:**

The online version contains supplementary material available at 10.1007/s13105-021-00789-x.

## Introduction

The increasing prevalence of obesity is associated with the rising incidence, progression, and worse prognosis of various human cancers including the breast, colon, and prostate [3, 4]. Additionally, obesity is also responsible of the more complicated management of patients with cancer [31]. Actually, the molecular basis linking obesity to carcinogenesis is not yet completely elucidated, but several mechanisms cooperate to create a functional relationship between these two pathologic states. Among others, metabolic alterations, hypoxia, and oxidative stress together with a deregulated secretion of adipokines have been recognized as risk factors of developing cancer [4, 19].

Adiponectin (Acrp30), the most abundant adipokine produced and secreted by adipose tissue, exerts protective functions on insulin resistance and inflammation by regulating peripheral energy metabolism as well as on cell proliferation, migration, and invasion [18, 23]. Furthermore, serum Acrp30 reduction is associated with metabolic diseases [23] and several types of cancers related to metabolism, like breast, lung, and colon cancers [14, 19].

On the other hand, obesity is associated also with an increase of leptin levels, an adipokine involved in the regulation of body weight and energy balance [16]. First discovered for its role in suppressing appetite and regulating energy expenditure, in vitro and in vivo studies have shown that beyond the metabolic functions, leptin is able to promote cell proliferation and migration and tumor growth [2]. Furthermore, serum levels of leptin are deregulated in metabolic and immune diseases and have been associated with different types of cancers [2, 27].

Previous research, through in vivo and in vitro studies, reported that Acrp30 and leptin may affect the behavior of cancer cells in an opposite manner [33], suggesting that the relation between these adipokines might be of value in cancer therapy and in the management of patient affected by several kinds of cancer.

Thyroid cancer (TC), accounting for the majority of endocrine malignancies, is suggested to be obesity-related; and, based on worldwide statistics, it is characterized by an increasing incidence in recent years [29]. The most prevalent subtype is papillary thyroid cancer (PTC) a well-differentiated carcinoma characterized by an indolent behavior and good outcome. Nevertheless, some PTC patients develop resistance to radioiodine therapy, metastases dissemination, and, consequently, an increased risk of death [5, 6, 32]**.**

The RAS/RAF/mitogen-activated protein kinase (MAPK) pathway has been described as one of the most important regulatory pathway involved in TC, and activating mutations of members of the MAPK signaling pathway are the main driver genetic lesions in TC [7].

Few data have been published about the regulation of Acrp30 levels in TC patients, with inconclusive results. It is also been reported that circulating leptin levels are also associated with thyroid carcinoma [1, 27]. Moreover, Cheng and colleagues reported that leptin is able to enhance migration of PTC cells [10, 11].

In this scenario, we have evaluated in vitro effects of Acrp30 and leptin in thyroid cancer cell lines. Interestingly, our results showed that Acrp30 reduces proliferation in papillary (BCPAP and K1) and anaplastic (CAL62) thyroid cancer cell lines. We also found that Acrp30 reduced migration and invasiveness of BCPAP and K1 cells, while leptin increased the motility phenotype of these cells. Additionally, since leptin is known to exhibit opposite Acrp30 actions, we evaluated the combined effect of these adipokines on malignant hallmarks of PTC transformation, and we found that Acrp30 and leptin have antagonizing effects on thyroid cancer cell proliferation and motility. This study demonstrates that both adipokines could represent intriguing biologic targets for thyroid cancer’s management.

## Materials and methods

### Cell cultures

The human papillary (K1 and BCPAP) and anaplastic (CAL62) thyroid cancer cell lines were cultured in Dulbecco’s modified Eagle’s medium (DMEM, Thermo Fisher Scientific, Waltham, MA) supplemented with 10% fetal bovine serum (FBS), L-glutamine, and penicillin/streptomycin (Thermo Fisher Scientific) at 37 °C in a humidified atmosphere of 5% CO_2_. The CAL62 were obtained from DSMZ (Deutsche Sammlung von Mikroorganismen und Zellkulturen GmbH, Germany), the K1 from ECACC (European Collection of Authenticated Cell Cultures, Sigma-Aldrich), and the BCPAP from primary source (N. Fabien, France).

### Cell viability assays

Cell viability was determined by 3-[4.5-dimethylthiazol-2-yl]-2.5-dipheniltetrazolium bromide (MTT) colorimetric assay according to the manufacturer’s instructions. Briefly, BCPAP and K1 (1 × 10^3^) cells were seeded in 96-well plates, incubated overnight in DMEM 5% FBS treated with different doses of Acrp30 (0.15, 1.5, 15 or 50 μg/ml) (Biovendor, Heidelberg, Germany), or treated with leptin (125 ng/ml) (Sigma-Aldrich, St. Louis, MO) or co-treated with Acrp30 (15 μg/ml) and leptin (125 ng/ml). As control, BCPAP and K1 cells were incubated in 5% FBS medium alone. After 24, 48, and 72 h of treatment, cells were stained with MTT reagent, and the absorbance at optical density (O.D.) 550 nm was measured using a microplate reader (Model 550, Ultramar Microplate Reader; Bio-Rad, Hercules, CA).

BCPAP (1 × 10^3^) cells were seeded in 96-well plates and, the day after, treated with Acrp30 (15 μg/ml) or leptin (125 ng/ml) or their combination or with dabrafenib (0.1 μM, Selleckchem, Munich, Germany), a selective reversible ATP inhibitor of mutant BRAF, for 48 hours. For co-treatments, cells were pre-incubated for 1 h with Acrp30 (15 μg/ml) or leptin (125 ng/ml) or their combination. Cell viability was monitored by MTT assay. As a negative control (NC), cells were incubated in 5% FBS and paired equal to 100.

In addition, trypan blue reagent (Bio-Rad) was used to measure cell viability. In brief, BCPAP and K1 (1 × 10^4^) cells were seeded in 24-well plates. The next day, cells were treated with Acrp30 (15 μg/ml) or with leptin (125 ng/ml) or the combination in DMEM containing 5% FBS. After 72 h, cells were collected and stained for 5 min with 0.4% trypan blue according to manufacturer’s instructions and counted using TC10^TM^ Automated Cell Counter (Bio-Rad).

### Wound healing assay

BCPAP or K1 (3 × 10^5^) cells were seeded in a 6-well plate in complete culture media and grown to confluence. The day after, cells were treated with 4 μg/ml of mitomycin (Sigma-Aldrich) for 2 h to inhibit cell proliferation, and then a wound was inflicted using a tip. After washing with phosphate-buffered saline (PBS), cells were incubated with various concentrations of Acrp30 (1.5 μg/ml, 15 μg/ml), leptin (125 ng/ml), or the combination in comparison to untreated cells. The same positions along the scratch wound were observed and photographed at different time points using an inverted-phase-contrast microscope (Nikon microscope TS100 fluorescence and video camera). The rate of wound closure was calculated with Cell^a^ Software (Olympus Biosystem Gmb, London, UK) and expressed as percentage of the closure.

### Matrigel Matrix invasion assay

Matrigel Matrix invasion assay was used to analyze the ability of cancer cells to invade extracellular matrix as previously described [25]. In brief, 1 × 10^5^ cells (BCPAP and K1) were resuspended in DMEM 5% FBS containing respectively Acrp30 (15 μg/ml) or leptin (125 ng/ml) or the combination and plated onto the upper well of filter of 8-μM pore size (Costar, Cambridge, MA) covered with Matrigel Matrix (Matrigel, BD Biosciences, San Jose, CA). Complete medium containing 10% FBS was added to the bottom chambers and used as the chemoattractant. Then, cells were allowed to invade for 24 h (K1 cells) or for 48 h (BCPAP cells) at 37 °C in a humidified atmosphere containing 5% CO_2_. Non-invading cells were removed by washing with PBS and by a cotton swab, while the invading cells were fixed with 11% glutaraldehyde (Sigma-Aldrich) for 30 min, colored in crystal violet solution, eluted, and quantified at O.D. 550 nm.

Additionally, BCPAP invading cells were also visualized and quantified under the microscope to manually count the cells that have invaded the Matrigel Matrix. To this aim, 5 × 10^4^ cells were suspended in DMEM 5% FBS containing respectively Acrp30 or leptin or the combination and allowed to invade the Matrigel. The day after, invading cells were fixed in glutaraldehyde, washed with PBS, and stained with blue-fluorescent 4′,6-diamidino-2-phenylindole dihydrochloride (DAPI, Sigma Aldrich) nucleic acid according to manufacture protocols. Cell counts were determined using a light microscope in five random fields. The mean number of invading cells was then normalized to untreated cells (NC) paired equal to 1.

### RNA extraction and quantitative real time-PCR

Total RNA was extracted from a panel of papillary (TPC-1, BCPAP, K1), anaplastic thyroid cancer cell lines (CAL62 and 8505C), and normal human immortalized thyroid follicular epithelial cells (Nthy-ori 3-1) by using TRIzol Reagent (Thermo Fischer Scientific). The RNA concentration was quantified by using fluorescence-based detection with Qubit 4 Fluorometer (Thermo Fischer Scientific).

One microgram of total RNA from each cell line was subjected to reverse transcription with SuperScript III First-Strand Synthesis SuperMix (Thermo Fisher Scientific) according to the manufacturer’s instructions.

Gene expression was performed in C1000 Touch Thermal Cycler (Bio-Rad) using iQ SYBR Green Supermix (Bio-Rad) with the following thermal cycling parameters: 95 °C, 3 min, followed by 40 cycles of denaturation (95 °C, 10 s), annealing (60 °C, 30 s), and elongation (72 °C, 30 s). GAPDH was used as housekeeping gene; fold changes were calculated with the following method: 2^−ΔΔCt^. Each reaction was performed in duplicate, and the data was extracted with Bio-Rad CFX Maestro version 1.0 (Bio-Rad).

Primer sequences used for q-RT-PCR are as follows:

- Leptin receptor fw = 5′- ’TTGTGCCAGTAATTATTTCCTCTT -3′
- Leptin receptor rev = 5′- CACACCAAAGAATGAAAAAGCTAT-3′
- AdipoR1 fw = 5′- ‘CACGCCATGGAGAAGATGG -3′
- AdipoR1 rev = 5′- ‘TCATATGGGATGACCCTGCAAC -3′
- AdipoR2 fw = 5′- TTTGCCACCCCTCAGTATCG -3′
- AdipoR2 rev = 5′- GGATGATTCCACTCAGGCCT -3′
- T-Cadherin fw = 5′- CCCTACATCGGCCACGTCAT -3′
- T-Cadherin rev = 5′- ATCGGTGGCTGGGTCATCTG -3′
- GAPDH fw = 5′- CATGGCCTTCCGTGTTCCTA -3′
- GAPDH rev = 5′- CCTGCTTCACCACCTTCTTGAT -3′

### Analysis of The Cancer Genome Atlas data collection

The expression level and the prognostic value of Acrp30, leptin, and their receptors (AdipoR1, AdipoR2, T-Cadherin, and leptin receptor) were analyzed in a large panel of human thyroid cancer patients deposited in The Cancer Genome Atlas (TCGA) data collection. To this aim, gene expression profiling interactive analysis (GEPIA) server (http://gepia.cancer-pku.cn/) was used to investigate the expression level of the indicated genes across thyroid carcinoma (*n* = 512) and normal thyroid tissues (*n* = 59) [30].

To investigate the relapse-free survival (RFS) and overall survival (OS) probabilities of the indicated genes in thyroid cancer patients, the Kaplan-Meier survival plots, indicating the hazard rate (HR) with 95% confidence interval and the log-rank *p* values, were obtained using the http://kmplot.com/analysis (https://kmplot.com/analysis/).

### Statistical analysis

Data are expressed as mean of replicates ± standard error of the mean. Statistical analyses were carried out using GraphPad Prism 6 software (La Jolla, CA). *p* Values were determined by Student’s unpaired *t* test (two-tailed). For multiple comparisons analysis, the one-way ANOVA followed by the Tukey multiple comparisons test was performed. None outliers have been removed prior to statistical analysis. A *p* value of < 0.05 was considered to indicate a statistically significant result.

## Results

### Expression of Acrp30 and leptin and their receptors in thyroid cancer cell lines and tissues

**Fig. 1:**
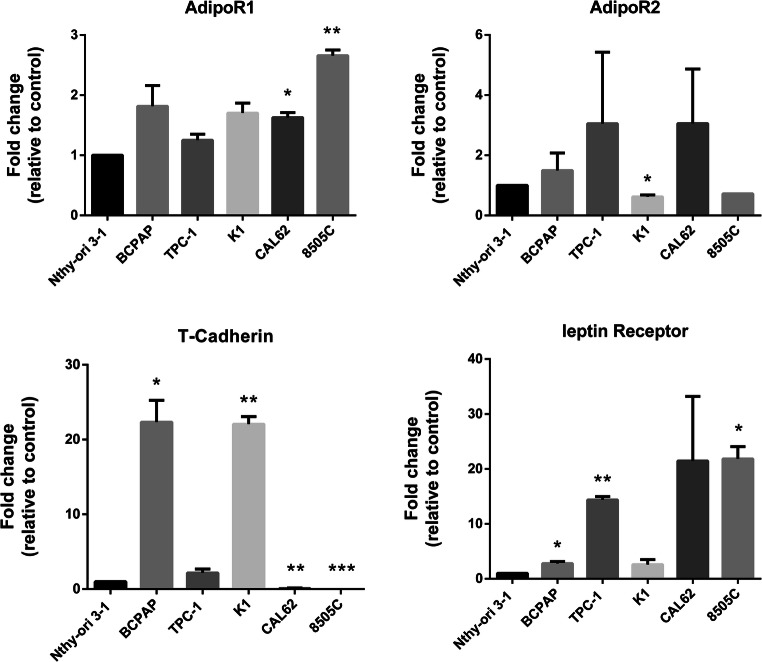
Expression level of Acrp30 and leptin receptors in human thyroid cancer cell lines. Quantitative RT-PCR was performed to analyze the expression level of Acrp30 receptors (AdipoR1, AdipoR2, and T-Cadherin) and of the leptin receptor in a panel of PTC (BCPAP, TPC-1, and K1) and ATC (CAL62 and 8505C) cell lines in comparison to normal immortalized human thyroid follicular epithelial cells (Nthy-ori 3-1). Each sample was normalized for the expression of GAPDH, used as endogenous control. Values are expressed as mean of replicates ± standard error of the mean (SEM). *p < 0.05; **p < 0.01; ***p < 0.001

We first investigated the expression of Acrp30 and leptin receptors in different human thyroid carcinoma cell lines including PTC (BCPAP, TPC-1, and K1) and ATC (CAL62 and 8505C) in comparison to normal human immortalized thyroid follicular epithelial cells (Nthy-ori 3-1). q-RT-PCR showed that the vast majority of the thyroid cancer cell lines tested, although with heterogeneity, expressed leptin and Acrp30 (AdipoR1, AdipoR2, and T-Cadherin) receptors when compared to Nthy-ori 3-1 (Fig. 1).

To further investigate the expression profiling of these genes also in thyroid cancer tissues, we interrogated the large dataset of PTC samples deposited in The Cancer Genome Atlas-Thyroid Cancer (TCGA-THCA) data collection [30]. Using GEPIA web server, we found a statistically significant overexpression of T-cadherin in thyroid tumor (*n* = 512) respect to normal thyroid tissues (*n* = 59) (Supplementary Fig. 1).

Finally, we investigated the prognostic value of Acrp30, of leptin, and of their receptors in thyroid cancer patients consulting the TCGA-THCA. Splitting patients in two different cohorts based of “low” and “high” median gene expression level of the indicating genes, Kaplan–Meier survival plot showed that low levels of leptin receptor are significantly correlated to overall survival but not with relapse-free survival (Supplementary Figs. 2 and 3). Furthermore, high level of Acrp30 is significantly correlated with relapse-free survival in thyroid cancer patients (Supplementary Fig. 3). These data underline the importance of monitoring the expression level of these genes in thyroid cancer patients.

### Acrp30 reduces thyroid cancer cell proliferation

Based on these results, successively, the effects of Acrp30 on cell viability were tested in K1 and BCPAP cell lines. Both cell lines were treated in 5% FBS with different doses of Acrp30 (0.15, 1.5, 15, 50 μg/ml) for 24, 48 and 72 h, and cell viability was evaluated using MTT assay. Supplementary Fig. 4 shows that Acrp30 reduced cell viability of K1 (A) and of BCPAP (B) cell lines in a time and dose dependent manner. In detail, the two highest doses (15 and 50 μg/ml) are already effective in reducing cell proliferation after 24 h of incubation in both cell lines. Since the dose of 15 μg/ml of Acrp30 is the lowest effective concentration, the next experiments were performed with this dose.

**Fig. 2:**
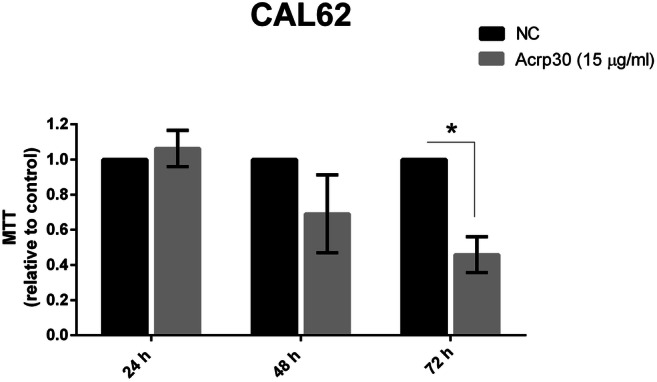
Acrp30 reduces the viability of ATC cell line. CAL62 cell line was treated with Acrp30 at the doses of 15 μg/ml for 24, 48, and 72 h. The effect on cell viability was evaluated by MTT assay. Untreated cells (grown in DMEM 5% FBS) were used as negative control (NC) and placed equal to 1. One-sample *t* test was performed, where the column means are significantly different than a hypothetical value (establishing 1 as the cutoff value). Values are expressed as mean of three different experiments ± standard error of the mean (SEM). *p < 0.05 versus NC

To uncover the effects of Acrp30 also in anaplastic thyroid carcinoma (ATC), an aggressive form of thyroid cancer, we treated CAL62 cell line with Acrp30 (15 μg/ml) and then evaluated cell proliferation after 24, 48 and 72 h from treatment. Figure 2 showed that 72 h post-treatment, Acrp30 has a significant impact on the reduction of proliferation.

### Leptin antagonizes Acrp30 effects on proliferation of papillary thyroid cancer cells

Previous studies reported that Acrp30 and leptin exhibit antagonizing effects in hepatocellular carcinoma [28]; thus, we have analyzed in our cell model system the effects of leptin treatment itself or in combination with Acrp30.

MTT assay was performed in PTC cell lines after 24, 48 and 72 h of treatments with Acrp30 and leptin alone or in combination. The dose of leptin 125 ng/ml was chosen according to Cheng et al. [10].

**Fig. 3:**
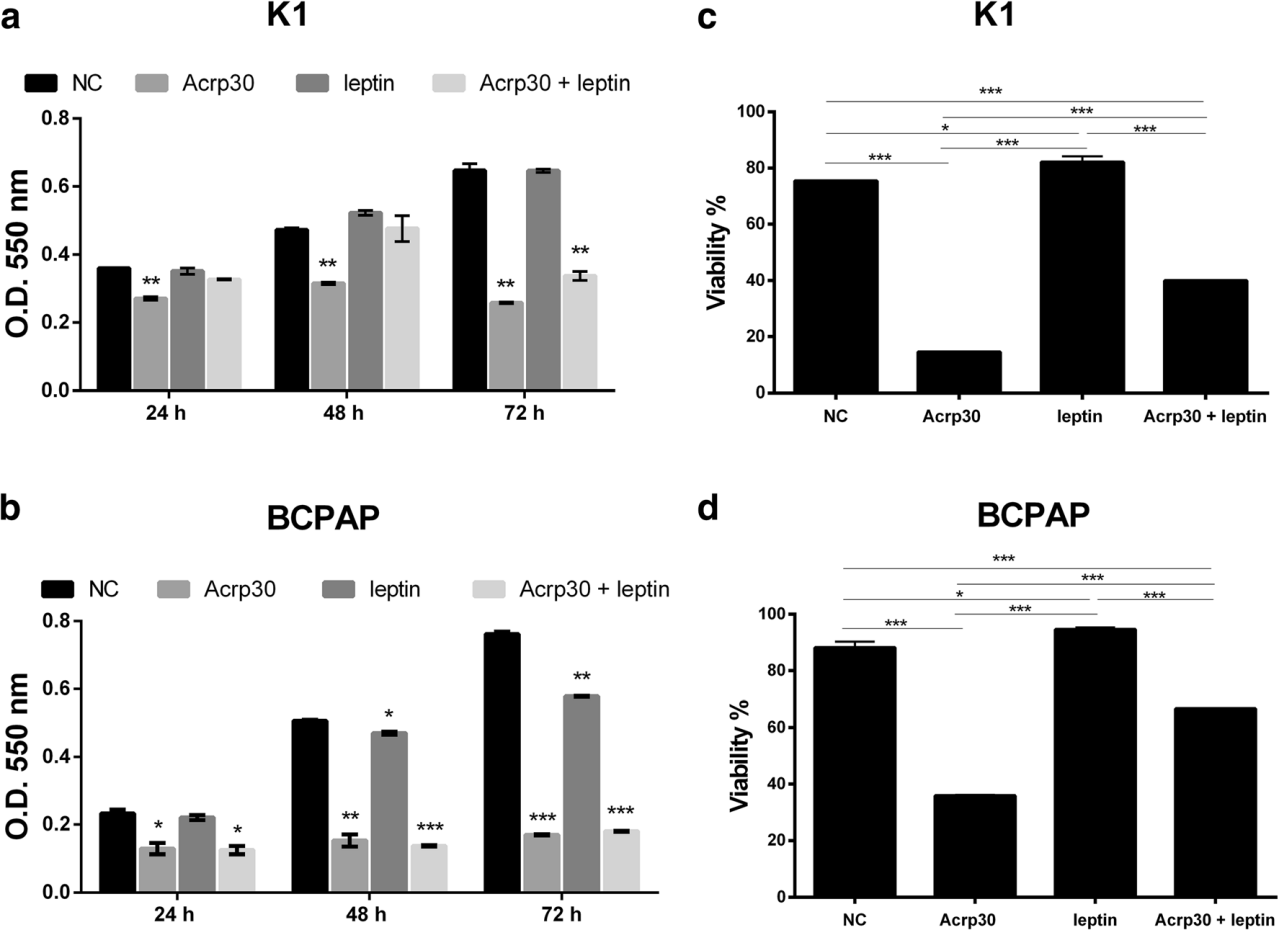
Leptin antagonizes Acrp30 effects on proliferation of PTC cells. **a,b** Cell viability was assessed by MTT assay in K1 (**a**) and in BCPAP (**b**) cell lines after 24, 48, and 72 h of treatment with Acrp30 (15 μg /ml) and leptin (125 ng/ml) alone or in combination. The differences were calculated with Student’s *t* test where *p < 0 05, **p < 0 01, ***p < 0 001 vs. untreated cells (NC). **c,d** The percentage of viability was also calculated by trypan blue assay in K1 (**c**) and in BCPAP (**d**) cell lines after 72 h of the treatments reported above. Viability values are calculated as ratio between trypan blue excluding cells (viable) and total cells and expressed as percentage. Untreated cells were used as negative control (NC). Values are expressed as mean of replicates ± standard error of the mean (SEM). Data were analyzed by one-way ANOVA followed by the Tukey multiple comparisons test. *p < 0.05; **p < 0.01; ***p < 0.001

As shown in Fig. 3, leptin treatment alone did not affect viability of K1 cells, whereas when used in combination, leptin can reduce Acrp30 inhibitory effects on proliferation (Fig. 3a). A similar response was observed performing the trypan blue assay. Indeed, as shown in Fig. 3c, at 72 h of treatment, in the combined treatment, leptin partially counteracts Acrp30 effects.

In BCPAP cells, in particular evident at the trypan blue assay, at 72 h of incubation, Acrp30 significantly reduced cell viability, while leptin partially counteracts Acrp30 effects in the combined treatment (Fig. 3d).

**Fig. 4:**
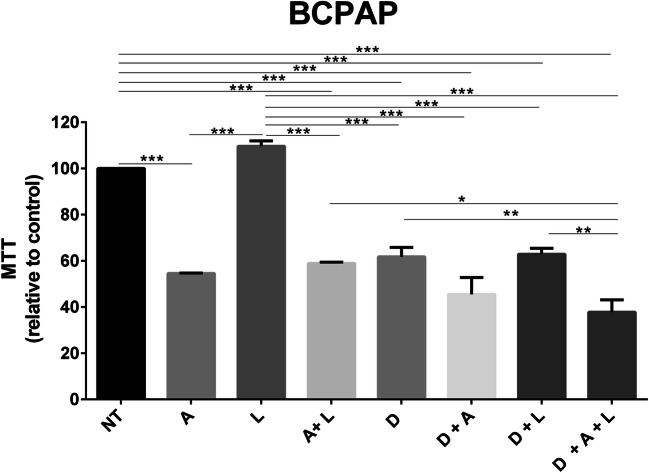
Effects of BRAF kinase inhibitor, leptin and of Acrp30 on cell viability. BCPAP cells were plated in 96-well plate and, the day after, incubated with Acrp30 (A, 15 μg/ml), leptin (L, 125 ng/ml) or with dabrafenib (D, 0.1 μM) or co-treated. After 48 h of incubation, cell viability was measured using MTT assay. Values are expressed as mean ± standard error of the mean (SEM) of one representative experiment performed in triplicate. Data were analyzed by one-way ANOVA followed by the Tukey multiple comparisons test. *p < 0.05; **p < 0.01; ***p < 0.001

Since it is reported in literature that the leptin effects are mediated by MAPK signaling pathway in thyroid cancer [11], here we evaluated whether the pharmacological inhibition of BRAF impairs the combined effects of the two adipokines on thyroid cancer cell proliferation. Thus, BCPAP cells were treated with Acrp30 (15 μg/ml), leptin (125 ng/ml), and dabrafenib (0.1 μM) alone or in combination; after 48 h, cell proliferation was evaluated by MTT assay. As shown in Fig. 4, dabrafenib rescued the antagonizing effects induced by leptin on Acrp30.

### Acrp30 and leptin exert antagonizing effects on papillary thyroid cancer cell migration

**Fig. 5:**
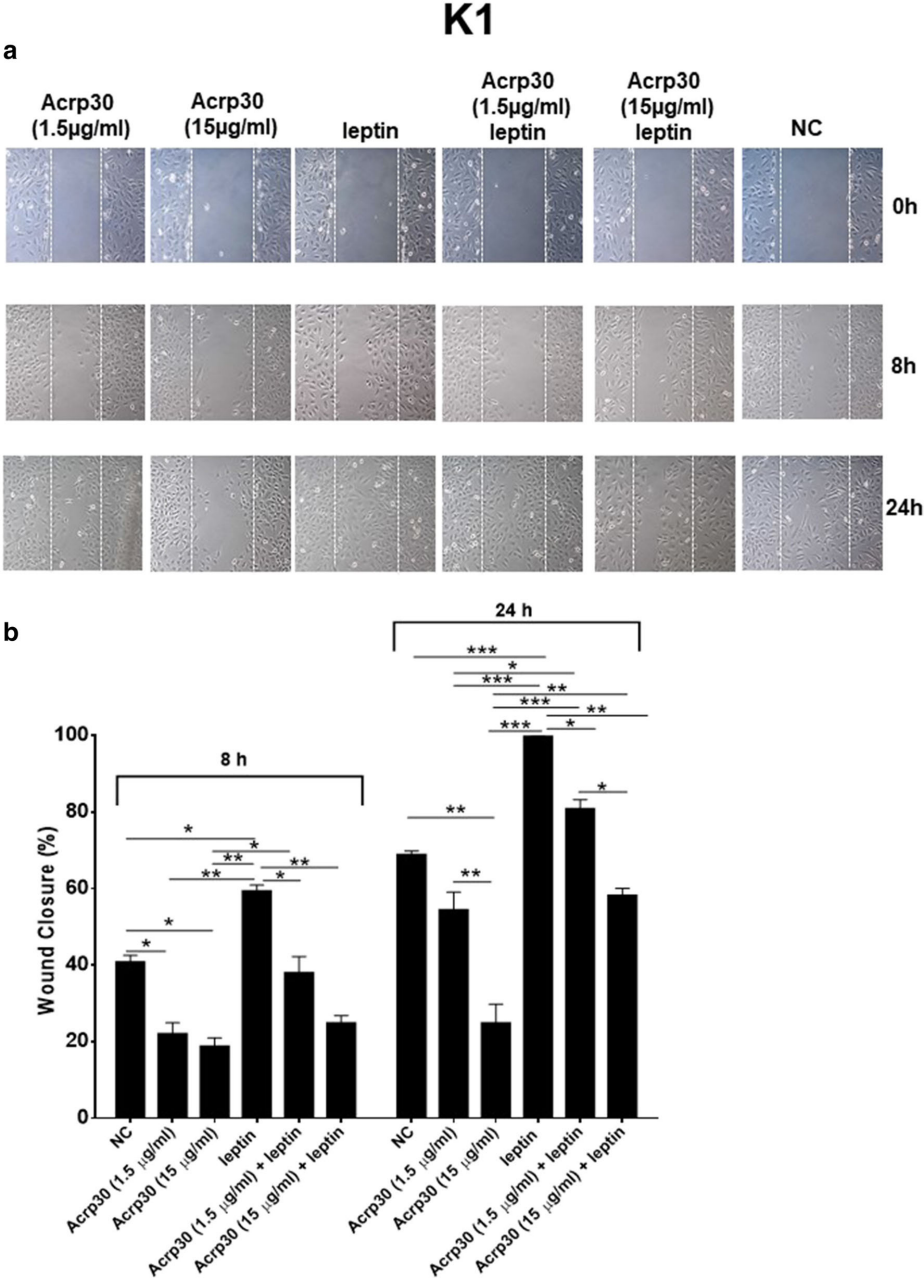
Acrp30 and leptin exert antagonizing effects on papillary thyroid cancer cell migration. K1 cells were treated with two different doses of Acrp30 (1.5 or 15 μg/ml) or leptin (125 ng/ml) alone or in combination and a scraped wound was afflicted. **a** Wound repairs were photographed immediately following the scratch (0 h), after 8 h, and at wound closure. Untreated cells, grown in DMEM 5% FBS, were used as a negative control (NC). Representative figures are shown from one of two independent experiments. **b** Wound closure was measured by calculating pixel densities in the wound area and expressed as percentage of wound closure ± standard error of the mean (SEM). The statistical analysis was evaluated using the one-way ANOVA test. *p < 0.05; **p < 0.01; ***p < 0.001

**Fig. 6:**
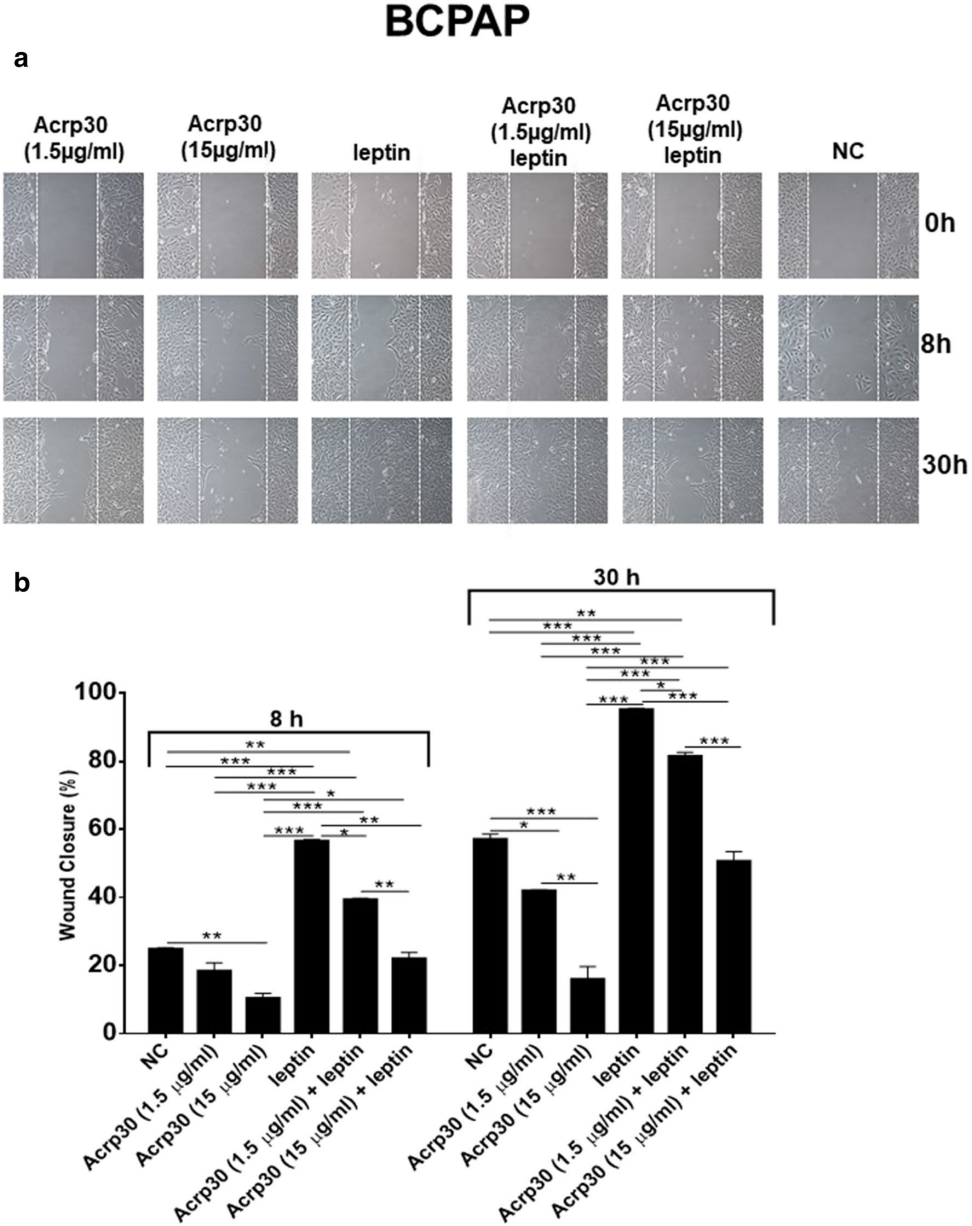
Acrp30 and leptin exert antagonizing effects on BCPAP cell migration. A scraped wound was inflicted on a confluent monolayer of BCPAP cells treated with the indicated doses of Acrp30, with leptin, alone, or in combination. Immediately following the scratch (0 h), after 8 and 30 h, cells were photographed under microscopy (**a**), and the closure of the gap was measured by calculating pixel densities in the wound area and expressed as percentage of wound closure ± standard error of the mean (SEM) (**b**). The statistical analysis was evaluated using the one-way ANOVA test. *p < 0.05; **p < 0.01; ***p < 0.001 versus NC

Several studies reported that Acrp30 and leptin might influence the motility and migration of cancer cells. Therefore, the PTC cells, K1 (Fig. 5) and BCPAP (Fig. 6), were subjected to a scraped wound and then treated with two different doses of Acrp30 (1.5 or 15 μg/ml) or with leptin (125 ng/ml), alone, or in combination. Acrp30 treatment significantly inhibited the migration of both cell lines in a dose-dependent manner in comparison to untreated cells. On the contrary, the treatment with leptin increased the migration ability of both cell lines. Interestingly, Acrp30 and leptin co-treatment rescued the effects of the single adipokines in a dose-dependent manner.

### Acrp30 and leptin exert antagonizing effects on papillary thyroid cancer cells invasion

**Fig. 7:**
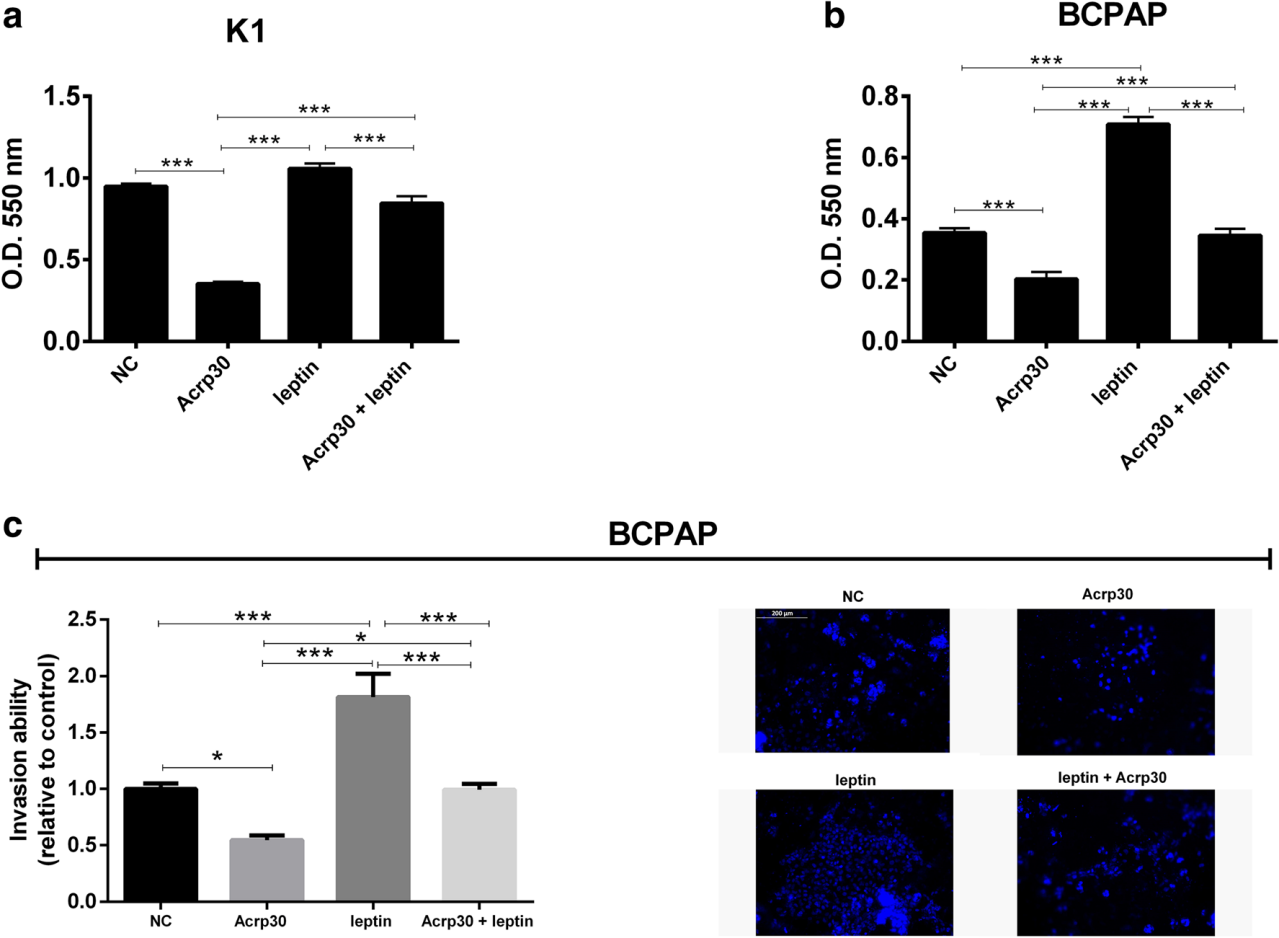
Acrp30 and leptin exert antagonizing effects on papillary thyroid cancer cells invasion*.* Both K1 and BCPAP cells were treated with Acrp30 (15 μg/ml), and leptin (125 ng/ml) alone or in combination and seeded in the upper chambers of transwell insert coated with Matrigel. K1 (**a**) and BCPAP (**b**) invading cells were stained with crystal violet and, after elution, quantized at O.D. 550 nm. **c** The number of BCPAP invading cells were colored with DAPI and counted at the microscope (× 10). Values are expressed as mean of replicates ± standard error of the mean (SEM). Data were analyzed by one-way ANOVA followed by the Tukey multiple comparisons test. *p < 0.05; **p < 0.01; ***p < 0.001

Invasion ability of thyroid cancer cells in response to Acrp30 or leptin treatment, alone, or in combination was analyzed by Matrigel Matrix invasion assay. K1 and BCPAP cell lines were treated with Acrp30 (15 μg/ml) and leptin (125 ng/ml) alone or in combination and seeded in the upper chambers of transwell insert coated with Matrigel. Invading cells, colored with crystal violet, were quantized after 24 h for K1 cells (Fig. 7a) and after 48 h for BCPAP cells (Fig. 7b). Moreover, invading BCPAP treated cells were also manually counted under the microscope after staining with DAPI (Fig. 7c left) and photographed (Fig. 7d). While Acrp30 treatment inhibited cell invasion, leptin increased invasion of both K1 and BCPAP compared to untreated cells. Interestingly, K1 and BCPAP cells co-treated with Acrp30 and leptin displayed the same invasion capability of untreated cells indicating that Acrp30 and leptin display antagonizing effects on thyroid cancer cell invasion (Fig. 7).

## Discussion

Several epidemiological studies have associated increased adiposity with increased incidence and/or progression and mortality of several malignancies [3, 4]. However, the mechanisms underlying the obesity-cancer relationship are still poorly understood. In this context, it is to note that adipose tissue participates to metabolic and/or inflammatory processes through the production of adipokines involved not only in immune responses but also in the regulation of energy metabolism, insulin sensitivity, and inflammation [4]. Among the adipokines, Acrp30 and leptin gained great attention since their serum levels have been found deregulated in different human cancers [13, 27]. Furthermore, in different cancer cell lines, Acrp30 exerts anti-proliferative and pro-apoptotic effects [17, 22, 24]. On the contrary, leptin stimulates angiogenesis and cell proliferation and downregulates anti-inflammatory cytokines [1, 2, 16].

Thyroid cancer (TC) is a tumor suggested to be associated with obesity [26]; additionally, a strict relationship between thyroid gland and adipokines was demonstrated by the altered expression of these proteins in several pathophysiological conditions [15].

Consistent evidence shows that in patients affected by thyroid cancer, the circulating levels of Acrp30 are deregulated compared to healthy controls, while the correlations between leptin and thyroid cancer are not clear. Mitsiades and colleagues showed that the circulating Acrp30 concentrations are significantly lower in TC patients than control subjects [21]. More recently, also in a prospective study, Dossus and colleagues suggested a negative association between the pre-diagnostic circulating level of Acrp30 in woman and TC risk, unlike the circulating level of leptin which appears to be not associated with TC risk [15]. Accordingly, Mele and colleagues showed that circulating Acrp30 levels were lower in patients with differentiated TC compared to subjects with benign thyroid diseases and healthy controls, while the circulating level of leptin was comparable between these groups [20]. Nevertheless, other studies reported that the serum leptin levels were higher in TC patients compared to control subjects and decreased following surgery [1, 27]. A recent meta-analysis highlights that leptin is significantly associated with TC risk [35].

Besides, the expression of Acrp30 receptors (AdipoR1, R2, and T-Cadherin) and leptin receptor are also correlated with several clinical-pathological features in TC patients. Indeed, Mitsiades reported that tissues and cell lines derived from human papillary thyroid carcinoma expressed Acrp30 receptors (AdipoR1 and AdipoR2) [21]. Additionally, Cheng and colleagues found that AdipoR1 and R2 are over-expressed in some PTC samples and their expression are inversely correlated with extrathyroidal invasion, multicentricity, and higher TNM stage, suggesting that their overexpression is associated with a better prognosis [12].

Several studies also reported that the expression of leptin and its receptor are associated with greater tumor size in Chinese PTC patients albeit with some differences in the results [9, 34].

In this scenario, here, we also investigated the prognostic value of Acrp30, leptin, and of their receptors in thyroid cancer patients consulting The Cancer Genome Atlas (TCGA-THCA) dataset [7], and we found that a better overall survival (OS) and relapse-free survival (RFS) are significantly correlated with low level of leptin receptor and with high level of Acrp30, respectively. These data are consistent with the role of these adipokines reported in literature; but unexpectedly, we also found that OS is significantly correlated with low level of AdipoR2 and with high level of leptin, while a better RFS is correlated with high level of leptin receptor in thyroid cancer patients. Consequently, even if the prognostic value of Acrp30, leptin, and their receptors remain controversial and further research are needed, our data underline the importance of monitoring the levels of these adipokines and their receptors in thyroid cancer patients.

Here, we also evaluated the effects of Acrp30 and leptin on the phenotype of TC cell lines. First, we have obtained evidence that Acrp30 has an anti-proliferative effect in papillary (K1 and BCPAP) and anaplastic (CAL62) TC cell lines. We also found that Acrp30 treatment has an anti-motility effect in PTC cells.

Additionally, this study unveils that Acrp30 and leptin have antagonizing effects on three important mechanisms at the basis of TC establishment and development: proliferation, migration, and invasion. We found that in BCPAP cells, the antagonizing effect of leptin against Acrp30 was more evident in the trypan blue assay than in the MTT assay, probably due to a perturbation of the cellular metabolic activity induced by leptin.

Coherently with our results, in literature, it is reported that Acrp30 is able to antagonize the oncogenic actions of leptin on hepatocellular carcinomas [28].

In line with our results, more recently, Celano and colleagues reported that leptin slightly increased cell proliferation of K1 cells but only at the dose of 500 ng/ml at 96 h-time point and increased cell motility [8]. While, consistent with our result, lower doses of leptin (125 ng/ml) does not impair proliferation in K1 cells. It is worth to point out that the dose of leptin of 125 ng/ml is above the physiological levels.

Likewise, Cheng and colleagues reported that the treatment with 125 ng/ml of leptin alone does not affect cell proliferation but increases migration of BCPAP and K1 thyroid cancer cells [11]. Additionally, the authors showed that this effect occurs through the activation of PI3K/AKT and MAPK, two signaling pathways that play a pivotal role in thyroid tumorigenesis [11]. In this context, we also found that the BRAF inhibitor, dabrafenib, an ATP-competitive inhibitor that inhibits mutant BRAF (V600E), rescued the antagonizing effects induced by leptin on Acrp30 confirming the dependence of this pathway on leptin effects.

Interestingly, in a previous pilot study, Cheng investigated the effects of leptin also in ATC (ARO), FTC (WRO), and PTC (CGTH-W3) cell lines. They found that leptin increased the migration of PTC cells but inhibited this ability in anaplastic and follicular cancer cells suggesting that leptin modulates cell migration of thyroid cancer cells in a cell type-specific manner. However, some of the cell lines used in the study were successively shown to be cross-contaminated or misidentified [10].

Here, we found that the different cell lines used show different sensitivity to the actions of Acrp30. This could be due to different genetic background or the different expression of adipokines receptors. Another possibility is that the differences in the cell lines may be due to their cellular origin; indeed, the BCPAP is a cell line derived from a PTC, K1 cell lines derives from a metastasis of PTC, while CAL62 cell line derives from an ATC.

Thus, our data sustain the role of adipokine in linking adipose tissue with thyroid cancer. Altogether, these data encourage deepening the research role of Acrp30 and leptin in TC as useful therapeutic targets and biomarkers for thyroid cancer.

## Supplementary Information

Supplementary Fig. 1 **Expression level of Acrp30, leptin and of their receptors in human thyroid cancer tissues.** The expression level of Acrp30 and of its receptors (AdipoR1, AdipoR2 and T-Cadherin) (**A**) and the expression level of leptin and its receptor (**B**) was observed in 512 thyroid cancer tissues in comparison to 59 normal thyroid tissues deposited by TCGA-THCA using GEPIA web server (http://gepia.cancerpku.cn). Box plot shows the expression of the indicated mRNA reposted as log2 (TPM + 1) transformed expression data. For the statistical analysis one-way ANOVA was performed, using disease state (Tumor or Normal) as variable for calculating differential expression. * p < 0.05. (JPG 80 kb)

Supplementary Fig. 2 **Overall survival (OS) probability of Acrp30, leptin and their receptors in thyroid cancer patients.** The prognostic value of Acrp30 and its receptors (**A**) and of leptin and leptin receptor (**B**) was evaluated in thyroid cancer patients. In each panel it is reported the Kaplan–Meier survival plot, with hazard ratio (HRs) and p-values (log-rank test) for two patient cohorts: red and black color indicated patients with high or low level of the genes respectively. (JPG 122 kb)

Supplementary Fig. 3 **Relapse-free survival (RFS) probability of Acrp30, leptin and their receptors in thyroid cancer patients.** Kaplan–Meier curves showed the RFS probability thyroid cancers for Acrp30 and its receptors (**A**) and of leptin and leptin receptor (**B**). Patients were split in two different cohorts: low (indicated by black color) and high (indicated by red color) on the base of the gene expression level. (JPG 127 kb)

Supplementary Fig. 4 **Acrp30 reduces viability of PTC cell lines in a time and dose-dependent manner.** Cell viability of K1 and of BCPAP cell lines (A and B, respectively) was assessed by MTT assay after 24, 48 and 72 hours of treatment with various doses of Acrp30 (0.15, 1.5, 15, 50 μg/ml). Untreated cells, grown in DMEM 5% FBS, were used as negative control (NC). Values are expressed as mean of replicates ± standard error of the mean (SEM). * p < 0.05; ** p < 0.01; *** p < 0.001 *versus* NC. (JPG 1019 kb)
