# Supplementary figures and images for: Adiponectin and leptin exert antagonizing effects on proliferation and motility of papillary thyroid cancer cell lines
Source: J Physiol Biochem. 2021 Feb 15;77(2):237–48. doi: 10.1007/s13105-021-00789-x (PMC8121733; doi:10.1007/s13105-021-00789-x)

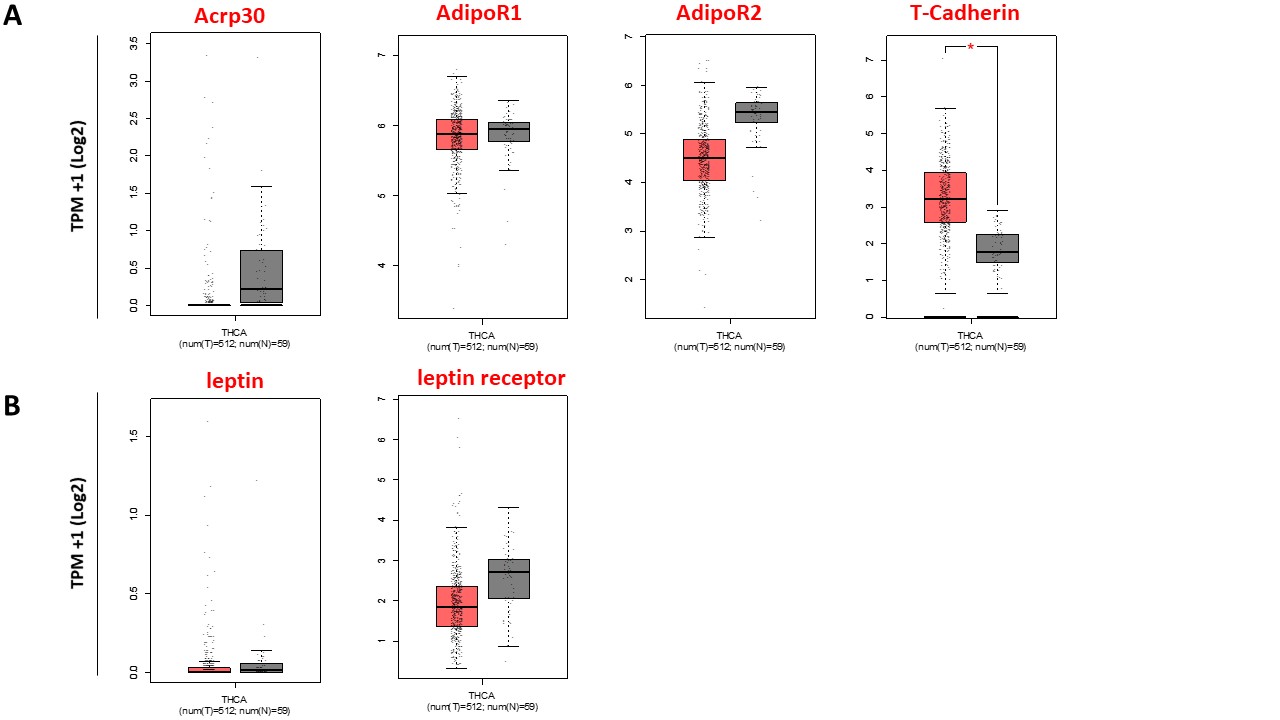

Supplement: Supplementary file 1 — Expression level of Acrp30, leptin and of their receptors in human thyroid cancer tissues. The expression level of Acrp30 and of its receptors (AdipoR1, AdipoR2 and T-Cadherin) (A) and the expression level of leptin and its receptor (B) was observed in 512 thyroid cancer tissues in comparison to 59 normal thyroid tissues deposited by TCGA-THCA using GEPIA web server (http://gepia.cancerpku.cn). Box plot shows the expression of the indicated mRNA reposted as log2 (TPM + 1) transformed expression data. For the statistical analysis one-way ANOVA was performed, using disease state (Tumor or Normal) as variable for calculating differential expression. * p < 0.05. (JPG 80 kb) [file 13105_2021_789_MOESM1_ESM.jpg]

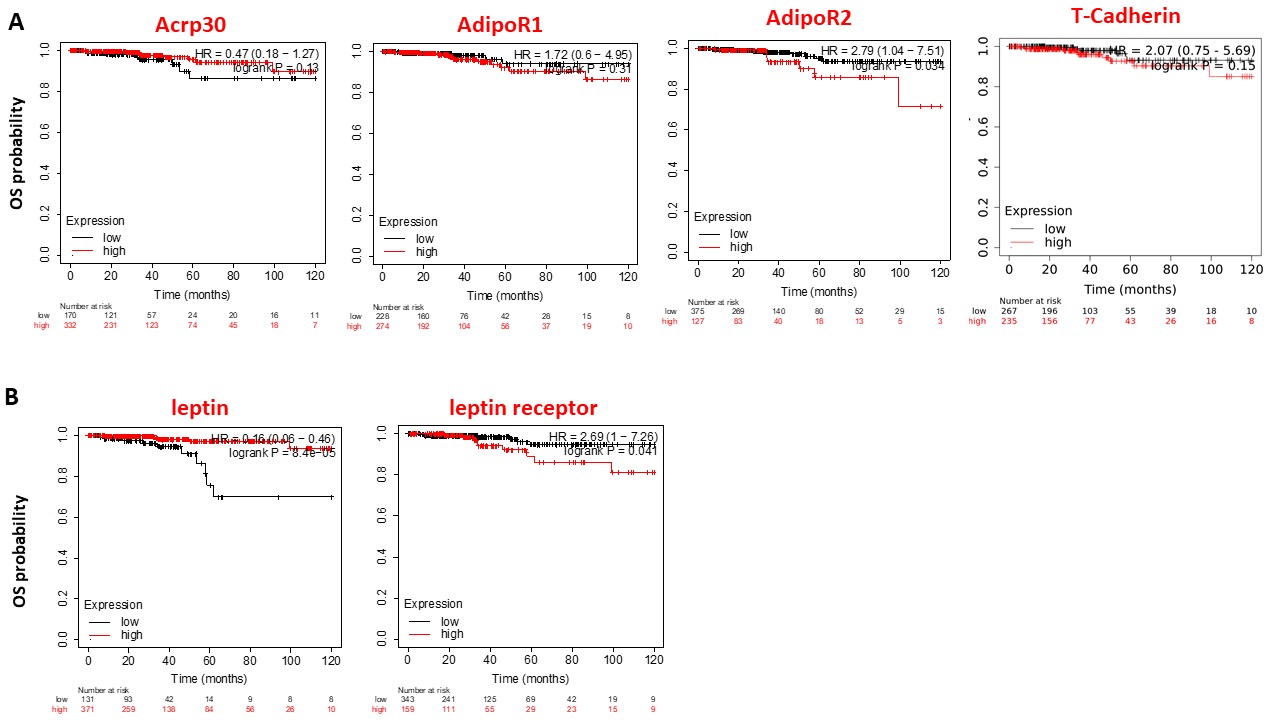

Supplement: Supplementary file 2 — Overall survival (OS) probability of Acrp30, leptin and their receptors in thyroid cancer patients. The prognostic value of Acrp30 and its receptors (A) and of leptin and leptin receptor (B) was evaluated in thyroid cancer patients. In each panel it is reported the Kaplan–Meier survival plot, with hazard ratio (HRs) and p-values (log-rank test) for two patient cohorts: red and black color indicated patients with high or low level of the genes respectively. (JPG 122 kb) [file 13105_2021_789_MOESM2_ESM.jpg]

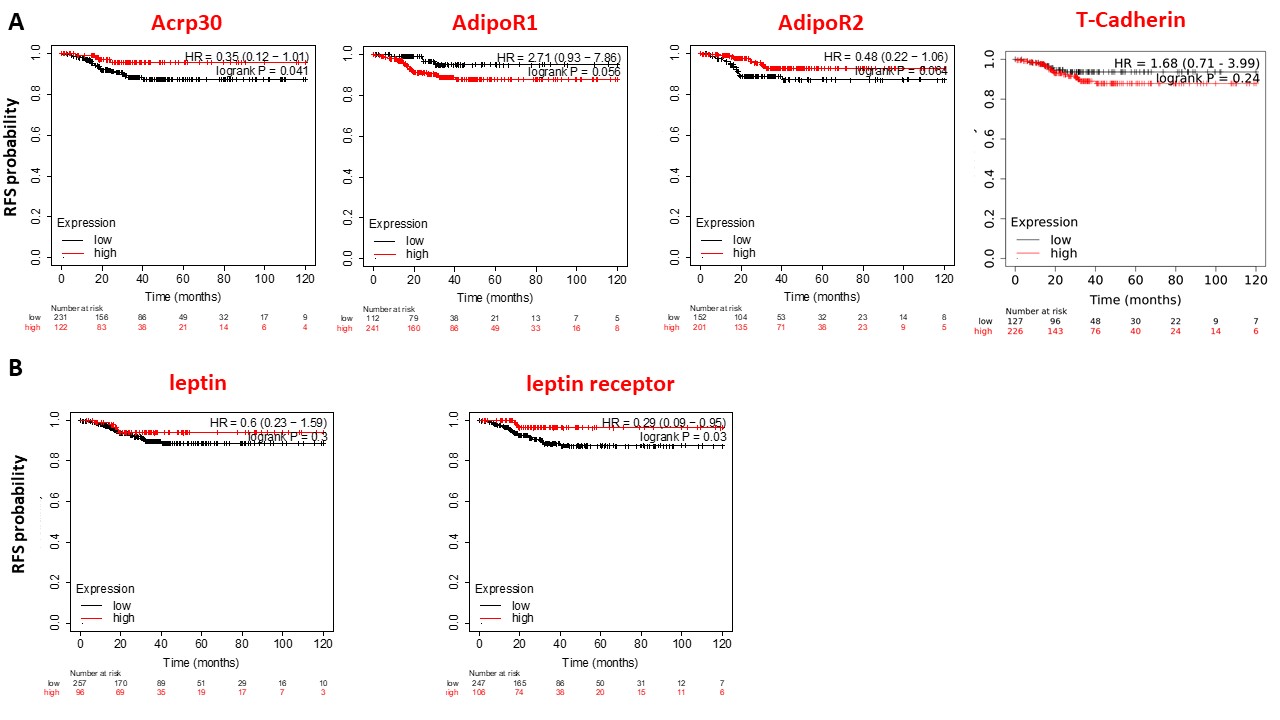

Supplement: Supplementary file 3 — Relapse-free survival (RFS) probability of Acrp30, leptin and their receptors in thyroid cancer patients. Kaplan–Meier curves showed the RFS probability thyroid cancers for Acrp30 and its receptors (A) and of leptin and leptin receptor (B). Patients were split in two different cohorts: low (indicated by black color) and high (indicated by red color) on the base of the gene expression level. (JPG 127 kb) [file 13105_2021_789_MOESM3_ESM.jpg]

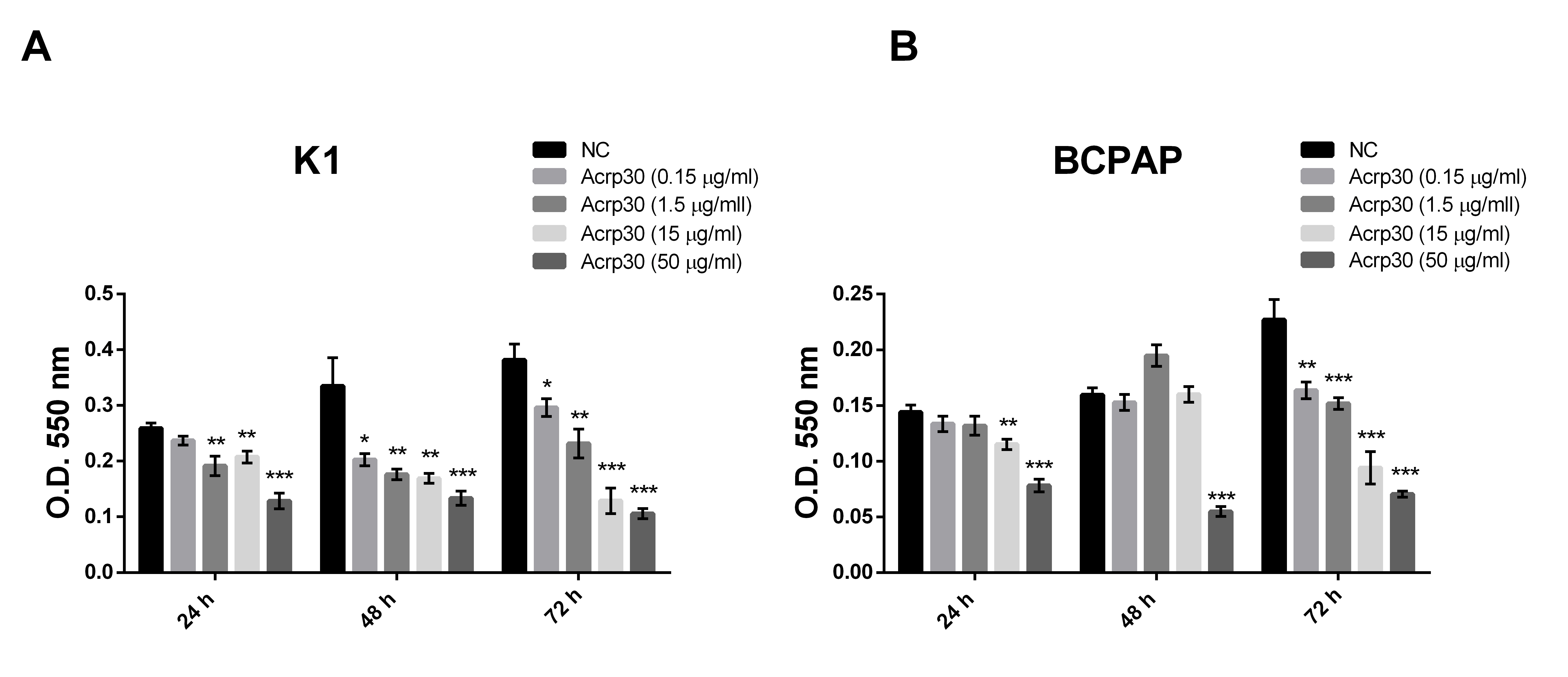

Supplement: Supplementary file 4 — Acrp30 reduces viability of PTC cell lines in a time and dose-dependent manner. Cell viability of K1 and of BCPAP cell lines (A and B, respectively) was assessed by MTT assay after 24, 48 and 72 hours of treatment with various doses of Acrp30 (0.15, 1.5, 15, 50 μg/ml). Untreated cells, grown in DMEM 5% FBS, were used as negative control (NC). Values are expressed as mean of replicates ± standard error of the mean (SEM). * p < 0.05; ** p < 0.01; *** p < 0.001 versus NC. (JPG 1019 kb) [file 13105_2021_789_MOESM4_ESM.jpg]
